# Trends of antiseizure medication utilization among pregnant people in four Canadian provinces from 1998 to 2023; a study from the Canadian mother-child cohort active surveillance initiative (CAMCCO)

**DOI:** 10.3389/fphar.2024.1469552

**Published:** 2024-11-12

**Authors:** Payam Peymani, Anick Berard, Brandace Winquist, Padma Kaul, Odile Sheehy, Alekhya Lavu, Christine Leong, Jamie Falk, Joseph A. Delaney, Kaarina Kowalec, Marcus Ng, Chelsea Ruth, Laila Aboulatta, Silvia Alessi-Severini, Roxana Dragan, Shelley Derksen, Olesya Barrett, Golnaz Shams, Sherif Eltonsy

**Affiliations:** ^1^ College of Pharmacy, Rady Faculty of Health Sciences, University of Manitoba, Winnipeg, MB, Canada; ^2^ Research Center, CHU Sainte-Justine, Montreal, QC, Canada; ^3^ Faculty of Pharmacy, University of Montreal, Montreal, QC, Canada; ^4^ College of Medicine, University of Saskatchewan, Saskatoon, SK, Canada; ^5^ Faculty of Medicine and Dentistry, University of Alberta, Edmonton, AB, Canada; ^6^ Canadian VIGOUR Center, University of Alberta, Edmonton, AB, Canada; ^7^ Department of Psychiatry, College of Medicine, Rady Faculty of Health Sciences, University of Manitoba, Winnipeg, MB, Canada; ^8^ Departments of Medicine and Epidemiology, University of Washington, Seattle, WA, United States; ^9^ Department of Medical Epidemiology and Biostatistics, Karolinska Institutet, Stockholm, Sweden; ^10^ Section of Neurology, Max Rady College of Medicine, University of Manitoba, Winnipeg, MB, Canada; ^11^ Department of Pediatrics and Child Health, Rady Faculty of Health Sciences, University of Manitoba, Winnipeg, MB, Canada; ^12^ Manitoba Centre for Health Policy, Winnipeg, MB, Canada; ^13^ Alberta Health Services, Edmonton, AB, Canada; ^14^ Pharmaceutical Sciences Research Center, Shiraz University of Medical Sciences, Shiraz, Iran; ^15^ Children’s Hospital Research Institute of Manitoba, Winnipeg, MB, Canada

**Keywords:** pregnancy, antiseizure medications, epilepsy, drug utilization, prescriptions, monotherapy, polytherapy, Canada

## Abstract

**Background:**

Epilepsy management during pregnancy is crucial for both the mother and fetus. The use of antiseizure medications (ASMs) during pregnancy requires careful consideration due to their potential effects on maternal and fetal health.

**Methods:**

This study analyzed trends in ASMs use among pregnant people in four Canadian provinces over 20 years (Manitoba, Saskatchewan, Alberta, and Quebec). Descriptive statistics were utilized to examine the characteristics of the population, with the frequency and patterns of ASM use estimated throughout each trimester. Linear regression models were developed to analyze yearly patterns of ASM utilization for the overall study population, as well as for people with and without epilepsy.

**Results:**

Among 1,317,141 pregnant individuals across four provinces, 0.7% had epilepsy. Of the total pregnancies, 1.7% (n = 22,783) were exposed to ASMs, comprising 4,392 from pregnant people with epilepsy (PPWE) and 18,391 from those without epilepsy (PPWOE). Results demonstrated varying trends in ASM usage between provinces, with an overall increase in usage among people without epilepsy in Manitoba, Saskatchewan, and Alberta. ASM use among PPWOE surged significantly in Manitoba (24.2–149.1 per 10,000 pregnant people), Saskatchewan (29.4–107.0 per 10,000), and Alberta (65.7–241.7 per 10,000) (*p* < 0.05). In Alberta, PPWE’s ASM exposure also rose, from 23.6 in 2008 to 43.0 per 10,000 pregnant people in 2021, while Quebec witnessed a decrease from 59.2 in 1998 to 45.5 per 10,000 pregnancies in 2015. Analysis of ASM use by trimester illustrated a substantial decline among PPWOE from 365 days pre-pregnancy to the third trimester in all provinces. ASM utilization by drug class showcased significant shifts, with second-generation ASMs experiencing a notable rise. Carbamazepine, once prominent, declined, making way for lamotrigine. Regional variations underscore diverse preferences, such as clonazepam’s sustained popularity in Manitoba and Quebec.

**Conclusion:**

The study identified increasing trends in ASM use, particularly the increased use of second-generation ASMs, and differences in prescription patterns for pregnant individuals with and without epilepsy. These findings reveal changing ASM use patterns, including increased second-generation ASM use and regional disparities, providing valuable insights into real-world prescription practices.

## 1 Introduction

Epilepsy is one of the most serious neurological conditions, with an estimated prevalence between 0.3% and 0.7% among pregnant people (Houben et al., 2022; Shouman et al., 2022). Antiseizure medications (ASMs) play a crucial role in achieving seizure control, but their use during pregnancy requires careful consideration due to potential risks and effects on maternal and fetal health. The management of epilepsy during pregnancy requires a delicate balance between minimizing uncontrolled seizures and avoiding potential risks of exposure to ASMs to ensure the safety and wellbeing of both the pregnant person and their developing fetus.

ASMs are required to treat epilepsy (Hochbaum et al., 2022; Shouman et al., 2022). Pregnant people with epilepsy (PPWE) are advised to continue their ASMs during pregnancy to minimize maternal and newborn adverse outcomes associated with epilepsy including preeclampsia, preterm labor, placental abruption, poor fetal growth, fetal death and maternal mortality (Kulaga et al., 2011; Margulis et al., 2019; Singal et al., 2020). In Canada, results of the study in the province of Québec published by Kulaga et al. (2011) found that 79.6% of PPWE received monotherapy, 5.8% polytherapy, and 14.6% without exposure to ASM (Kulaga et al., 2011). The goal of treatment with ASMs during pregnancy is to optimize seizure control with medications with the least adverse outcomes to reduce the risk of neurodevelopmental disorders and malformations (Shouman et al., 2022; Cohen et al., 2023; Vajda et al., 2023). With the development of newer generation ASMs and the surge in their consumption for off-label use for mood disorders, anxiety disorders, neuropathic and chronic pain, migraine prophylaxis, and movement disorders during pregnancy, there has been an increase in the use of ASMs on a population basis, including among PPWE (Meador et al., 2018; Alsfouk et al., 2022). A study published in CMAJ in 2016 found that 40% of pregnancies in Canada were unplanned, indicating that these pregnancies may inadvertently be exposed to these medications (Hochbaum et al., 2022; Cohen et al., 2023; Vajda et al., 2023).

Studies of ASM prescribing in pregnant people have documented reduced prescribing of old-generation ASMs (Lavu et al., 2023a), with concurrent increases in the prescribing of the new generation ASMs (Cohen et al., 2020; Alsfouk et al., 2022; Hochbaum et al., 2022; Shouman et al., 2022). The studies demonstrate a significant shift from poly to monotherapy and from the old generation and older ASMs to the newer ASMs in PPWE (Kinney et al., 2018; Meador et al., 2018; Alsfouk et al., 2022; Hochbaum et al., 2022; Shouman et al., 2022).

Previously, using data from Manitoba, we showed a significant rise in ASM use among pregnant people without epilepsy (PPWOE) and a reduction in old ASMs generation (including valproic acid and carbamazepine) use followed by a rise in new generation ASMs (including levetiracetam and lamotrigine) use (Shouman et al., 2022). However, there have been limited population-based studies describing the use of ASMs in pregnant people across other Canadian provinces, particularly for newer agents. Recent long-term real-world data on ASMs utilization are lacking. Data on medication use patterns, alongside data on treatment effectiveness and safety in PPWE, can inform the development of evidence-based clinical practice guidelines. This will help healthcare professionals make informed decisions about prescribing medications for epilepsy in this population. (Kulaga et al., 2011; Yeh et al., 2017; Kinney et al., 2018; Margulis et al., 2019; Singal et al., 2020).

This study aimed to analyze the long-term trends of the utilization of ASMs in large cohorts of pregnant people from four Canadian provinces (Manitoba, Saskatchewan, Alberta, and Quebec) over 20 years (1998–2023) with special consideration for ASMs used throughout pregnancy, each trimester, and different generations of ASMs.

## 2 Materials and methods

### 2.1 Study setting and databases

This retrospective population-based cohort study utilized data from the Canadian Mother-Child Cohort (CAMCCO) (http://www.motherchildcohort.ca/) to investigate trends in ASM prescription among all pregnant individuals (Bérard et al., 2022). More specifically, the study cohort consisted of four harmonized pregnancy cohorts from Manitoba (University of Manitoba, Manitoba Center for Health Policy); Alberta (University of Alberta); Saskatchewan (University of Saskatchewan, Saskatchewan Health Quality Council), and Quebec (University of Montreal, CHU Ste-Justine) (Bérard et al., 2022). These four cohorts were built with the linkage of large provincial databases that included physician visits, hospital, medications, and sociodemographic real-world data and allowed us to conduct longitudinal follow-up and comprise detailed information on medication exposure. A detailed breakdown of data sources by province, including their start dates and any variations in data collection methods, can be found in the CAMCCO paper (Bérard et al., 2022).

The medical service databases include detailed information on maternal age, welfare status, place of residence (urban vs. rural); and all medical services such as medical and emergency visits history, hospital admissions, diagnoses, and procedures done by physicians and nurse practitioners using the International Classification of Diseases (ICD-9 and ICD-10) (Bérard et al., 2022). Furthermore, medical databases contain information that allows for the calculation of age and geographic location (urban vs. rural).

The prescription drug databases use Anatomical Therapeutic Chemical (ATC) classification and include prescriptions filled for the entire population, including drug name, quantity, dosage, duration of the prescription and dispensation date, except for the Quebec province, where drug data is limited to individuals covered by a public drug insurance plan. Unprescribed and unfilled medication OTC (over the counter) medications) or in-hospital medications were not available. Databases details, CAMMCO infrastructure, and Linkage procedures within each province can be found in the CAMCCO paper (Bérard et al., 2022) The validity of the prescription drug file’s data for capturing medication exposure has been confirmed at the Quebec site through highly positive predictive value (PPV) and negative predictive value (NPV). This indicates the data’s reliability in reflecting true medication use within this large database (Zhao et al., 2017).

The hospitalization databases (Discharge Abstract Database and National Ambulatory Care Reporting System) include all acute care hospitalizations, day surgery, outpatient and emergency visits.

Birth databases, which vary by province, provide demographic details about individuals, including information such as maternal marital status, birth weight of baby, and gestational age for live births and stillbirths. In Saskatchewan, birth information is obtained from delivery abstracts for in-hospital births, as access to Vital Statistics Birth Certificate data is not available. This approach captures data for approximately 99% of births in the province, given the high in-hospital birth rate. These vital data sources have been cross-referenced with medical charts and determined to be both complete and reliable for Manitoba, Quebec, and Saskatchewan (Zhao et al., 2017). In Alberta, pregnancy cohort is built using abstracted hospital records and perinatal health program data that includes both in-hospital and out-of-hospital births. Birth records are cross-referenced with Vital Statistics Birth data and provincial healthcare insurance plan registry.

### 2.2 Study population

We captured all diagnosed pregnancies for people living in Manitoba (1998–2021), Alberta (2008–2021), Saskatchewan (1998–2023) and Quebec (1998–2015) where the cohort only covers pregnant people insured by the public medication insurance, and thus included data from approximately 30% of Quebec pregnant people. Women are followed from the beginning of pregnancy, defined as the first day of the last menstrual period, until the end of pregnancy (planned or spontaneous abortion, or delivery). The end of pregnancy is determined using data on gestational age, validated against patient charts. Pregnant individuals are identified in the databases through prenatal visits or pregnancy-related procedures such as ultrasounds or amniocentesis. A person was considered to have epilepsy if they have one (or more) hospitalizations or physician billing visits for epilepsy (ICD-9 = 345 and ICD-10-CA = G40/G41) during the 5 years prior to delivery (except for Qc site, during the 1 year prior) (Fisher et al., 2014; Tu et al., 2014; Leong et al., 2016). Pregnancies ending before 1 January 1998, with deliveries that did not result in a live or stillbirth, pregnancies that were completed before 20 weeks of gestation, were exposed to proven teratogenic medication (Eltonsy et al., 2016), or did not have health insurance coverage 5 years before, were excluded from the study population. Four cohorts of pregnant people were generated: (1) exposed pregnant people with epilepsy, (2) exposed pregnant people without epilepsy, (3) unexposed pregnant people with epilepsy, and (4) unexposed pregnant people without epilepsy. We used postal codes from the Canada Census to distinguish urban from rural regions, and census data for income quintiles based on ranges of mean household income and grouped into five categories with each quintile assigned to approximately 20% of the population.

### 2.3 Antiseizure medications use

ASM utilization was identified using the ATC classification (N03A) (Supplementary Table S1). Exposure to an ASM regimen during pregnancy was classified as first trimester, second trimester, third trimester, and anytime during the pregnancy (Supplementary Table S2). Exposure to an ASM was defined as having at least one prescription within the designated exposure window or a prescription prior to the start of the exposure period, but with a duration that overlaps with the exposure window.

### 2.4 Comorbidities definitions

The following comorbidities were included in the descriptive statistics: diabetes, mood and anxiety disorders, personality disorders, pain, schizophrenia, and hypertension were defined according to (ICD-9-CM and ICD-10-CA) and ATC code classification (Supplementary Table S3).

### 2.5 Statistical analysis

A standardized and harmonized protocol was implemented, encompassing diagnosis and medication codes, statistical coding, algorithms, and modeling. This protocol facilitated database linkages, follow-up, and the identification of variables such as pregnancy, trimester, medication exposure, and the mother-child link in a consistent manner across all provinces.

Descriptive statistics were utilized to examine the characteristics and comorbidities of people such as mood disorders, diabetes, and hypertension outlined in Supplementary Table S3. The frequency and patterns of ASM usage were estimated throughout pregnancy, including each trimester. An assessment of annual ASM usage trends was conducted for the overall study population, as well as for people with epilepsy and those without epilepsy. Linear regression models were developed to analyze the yearly patterns of ASM utilization, both overall and for specific ASMs, within each group of pregnant people. The analysis spanned data from 1997 to 2023, considering the availability of certain medications starting from 1997, and data is reported as numbers per 10,000 pregnancies. Statistical significance was defined as a *p*-value of ≤0.05. All statistical analyses were performed using SAS software, version 9.4 (SAS Institute Inc., Cary, NC).

## 3 Results

**FIGURE 1:**
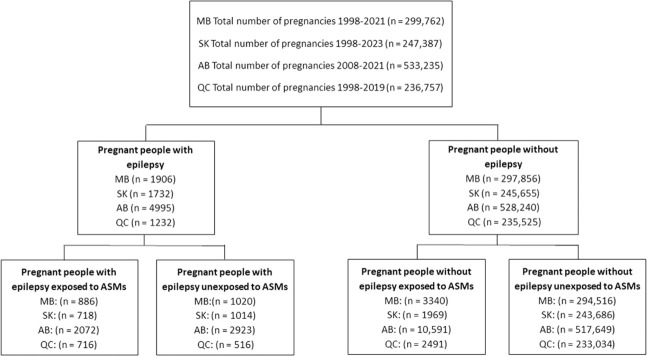
Study flowchart: MB, Manitoba; SK, Saskatchewan; AB, Alberta; QC, Quebec.

The individual prescription patterns for a total of 1,317,141 pregnancies were analyzed including 22.8% (n = 299,762) from Manitoba, 18.8% (n = 247,387) from Saskatchewan, 40.5% (n = 533,235) from Alberta, and 18.0% (n = 236,757) from Quebec. In the study population, 0.7% (n = 9,865) had epilepsy while 99.3% (n = 1,307,276) were PPWOE. Among these pregnancies, 1.7% (n = 22,783) were exposed to ASMs during their pregnancy (4,392 from PPWE and 18,391 from PPWOE) (Figure 1).

The characteristics of study cohorts are described in Table 1. The mean age of pregnant people at the start of their pregnancy was 28 years. PPWOE who were exposed to ASMs had the highest rate of comorbidities including hypertension, diabetes, mood and anxiety disorders, schizophrenia, and personality disorder in all four provinces, in comparison to other groups. One exception was the greater diagnosis of pain among unexposed PPWE compared to exposed PPWOE in Quebec which were 13.6% and 11.4% (*p* < 0.05), respectively.

**TABLE 1:** Characteristics of the study population by group.

	Manitoba				Saskatchewan				Alberta				Quebec			
	Exposed		Unexposed		Exposed		Unexposed		Exposed		Unexposed		Exposed		Unexposed	
	PPWE	PPWOE	PPWE	PPWOE	PPWE	PPWOE	PPWE	PPWOE	PPWE	PPWOE	PPWE	PPWOE	PPWE	PPWOE	PPWE	PPWOE
Total, N (%)	886 (0.3)	3,340 (1.1)	1,020 (0.3)	294,516 (98.2)	718 (0.3)	1,969 (0.8)	1,014 (0.4)	243,686 (98.5)	2072 (0.4)	10,591 (2.0)	2,923 (0.5)	517,649 (97.1)	716 (0.3)	2,491 (1.1)	516 (0.2)	233,034 (98.4)
Mean age Pregnant Person	28.1	29.5	26.7	28.1	27.4	28.7	25.2	27	28.6	29.3	27.4	29.2	27.5	29.8	27.4	28.2
Income quartiles, N (%)																
Q1 (Lowest income)	269 (30.4)	1,398 (41.9)	321 (31.5)	75,835 (25.7)	221 (30.8)	657 (33.4)	354 (34.9)	64,450 (26.4)	262 (12.6)	1,274 (12.0)	360 (12.1)	83,770 (16.2)	NA			
Q2	225 (25.4)	722 (21.6)	210 (20.6)	63,355 (21.5)	120 (16.7)	392 (19.9)	217 (21.4)	44,680 (18.3)	351 (16.9)	1,639 (15.5)	496 (16.7)	96,000 (18.5)				
Q3	177 (20)	527 (15.8)	210 (20.6)	54,446 (18.5)	119 (16.6)	323 (16.4)	160 (15.8)	39,556 (16.2)	372 (18.0)	1940 (18.3)	512 (17.2)	100,370 (19.4)				
Q4	128 (14.4)	367 (11)	179 (17.5)	54,027 (18.3)	120 (16.7)	283 (14.4)	147 (14.5)	45,664 (18.7)	428 (20.7)	2,188 (20.7)	645 (21.7)	102,296 (19.8)				
Q5 (Highest income)	81 (9.1)	315 (9.4)	97 (9.5)	46,032 (15.6)	90 (12.5)	215 (10.9)	92 (9.1)	33,627 (13.8)	588 (28.4)	3,085 (29.1)	839 (28.2)	115,424 (22.3)				
Area of Residence, N (%)																
Rural*	398 (44.9)	1,326 (39.7)	417 (40.9)	138,931 (47.2)	186 (25.9)	514 (26.1)	347 (34.2)	81,019 (33.2)	576 (27.8)	3,245 (30.6)	856 (28.8)	137,732 (26.6)	133 (18.6)	446 (17.9)	81 (15.7)	41,212 (17.7)
Urban*	488 (55.1)	2014 (60.3)	603 (59.1)	155,585 (52.8)	517 (72.0)	1,430 (72.6)	657 (64.8)	15,9624 (65.5)	1,496 (72.2)	7,345 (69.4)	2,120 (71.2)	380,005 (73.4)	583 (81.4)	2,045 (82.1)	435 (84.3)	191,822 (82.3)
Comorbidities N (%)																
HTN	37 (4.2)	304 (9.1)	32 (3.1)	4,882 (1.7)	27 (3.8)	151 (7.7)	22 (2.2)	3,883 (1.6)	79 (3.8)	628 (5.9)	75 (2.5)	6,859 (1.3)	16 (2.2)	137 (5.5)	17 (3.3)	2,814 (1.2)
DM	32 (3.6)	279 (8.4)	39 (3.8)	9,352 (3.2)	25 (3.5)	146 (7.4)	26 (2.6)	7,382 (3.0)	93 (4.5)	602 (5.7)	102 (3.4)	17,233 (3.3)	12 (1.7)	68 (2.7)	8 (1.6)	3,266 (1.4)
MAD	212 (23.9)	2,137 (64)	225 (22.1)	31,420 (10.7)	140 (19.5)	1,078 (54.7)	204 (20.1)	23,990 (9.8)	599 (28.9)	5,786 (54.6)	664 (22.3)	54,762 (10.6)	119 (16.6)	1,160 (46.6)	75 (14.5)	14,689 (6.3)
SCZ	7 (0.8)	48 (1.4)	S (−)	296 (0.1)	s (.)	23 (1.2)	s (.)	134 (0.1)	17 (0.8)	136 (1.3)	21 (0.7)	403 (0.1)	4 (0.6)	81 (3,3)	2 (0.4)	341 (0.2)
PD	13 (1.5)	133 (4)	12 (1.2)	917 (0.3)	s (.)	56 (2.8)	9 (0.9)	289 (0.1)	66 (3.2)	400 (3.8)	46 (1.6)	1,246 (0.3)	39 (5.5)	273 (11.0)	13 (2.5)	1,716 (0.7)
Pain	104 (11.7)	707 (21.2)	136 (13.3)	16,298 (5.5)	82 (11.4)	327 (16.6)	136 (13.4)	13,135 (5.4)	415 (20.0)	2,719 (25.7)	502 (16.8)	37,325 (7.2)	65 (9.1)	283 (11.4)	70 (13.6)	11,696 (5.0)
Birth Status N (%)																
Stillborn	S (−)	34 (1)	6 (0.6)	1983 (0.7)	7 (0.9)	34 (1.7)	10 (0.9)	1767 (0.7)	13 (0.6)	125 (1.2)	21 (0.7)	2,684 (0.5)	4 (0.6)	11 (0.4)	4 (0.8)	731 (0.3)
Singleton	862 (97.3)	3,244 (97.1)	1,000 (98)	286,568 (97.3)	704 (98.1)	1938 (98.4)	1,002 (98.8)	240,328 (98.6)	2034 (98.2)	10,390 (98.1)	2,924 (98.2)	508,642 (98.2)	705 (98.5)	2,473 (99.3)	512 (99.2)	231,094 (99.2)
Multiple births	24 (2.7)	96 (2.9)	20 (2)	7,948 (2.7)	14 (1.9)	31 (1.6)	12 (1.2)	3,358 (1.4)	38 (1.8)	200 (1.9)	52 (1.8)	9,122 (1.8)	11 (1.5)	18 (0.7)	4 (0.8)	1940 (0.8)

PPWE, pregnant people with epilepsy; PPWOE, pregnant people without epilepsy; N, number; SD, standard deviation; SES, socioeconomic status; HTN, hypertension; DM, diabetes mellitus; MAD, mood and anxiety disorders; SCZ, schizophrenia; PD, personality disorder; S, suppressed (Values ≤ 5 were suppressed).

* Source: Statistics Canada Postal Code Conversion File and/or Postal Codes by Federal Ridings File and/or Postal Code Conversion File Plus (November 2019) based on data licensed from Canada Post Corporation.

In Manitoba and Saskatchewan, the greatest percentage of pregnancies in all four groups belonged to the lowest income quintile. This distribution in Alberta is similar even for the unexposed group without epilepsy, suggesting it reflects the overall population distribution of income quartiles in the province. In the Quebec province, the greatest proportion of pregnant people were in the medium-low-income quantile. In Alberta, all four groups of pregnant people, including those unexposed without epilepsy, were mostly in the highest income quintile. This distribution is consistent with the overall population distribution of income quintiles in the region, indicating that the observed pattern reflects the broader socioeconomic status of the population.

**FIGURE 2:**
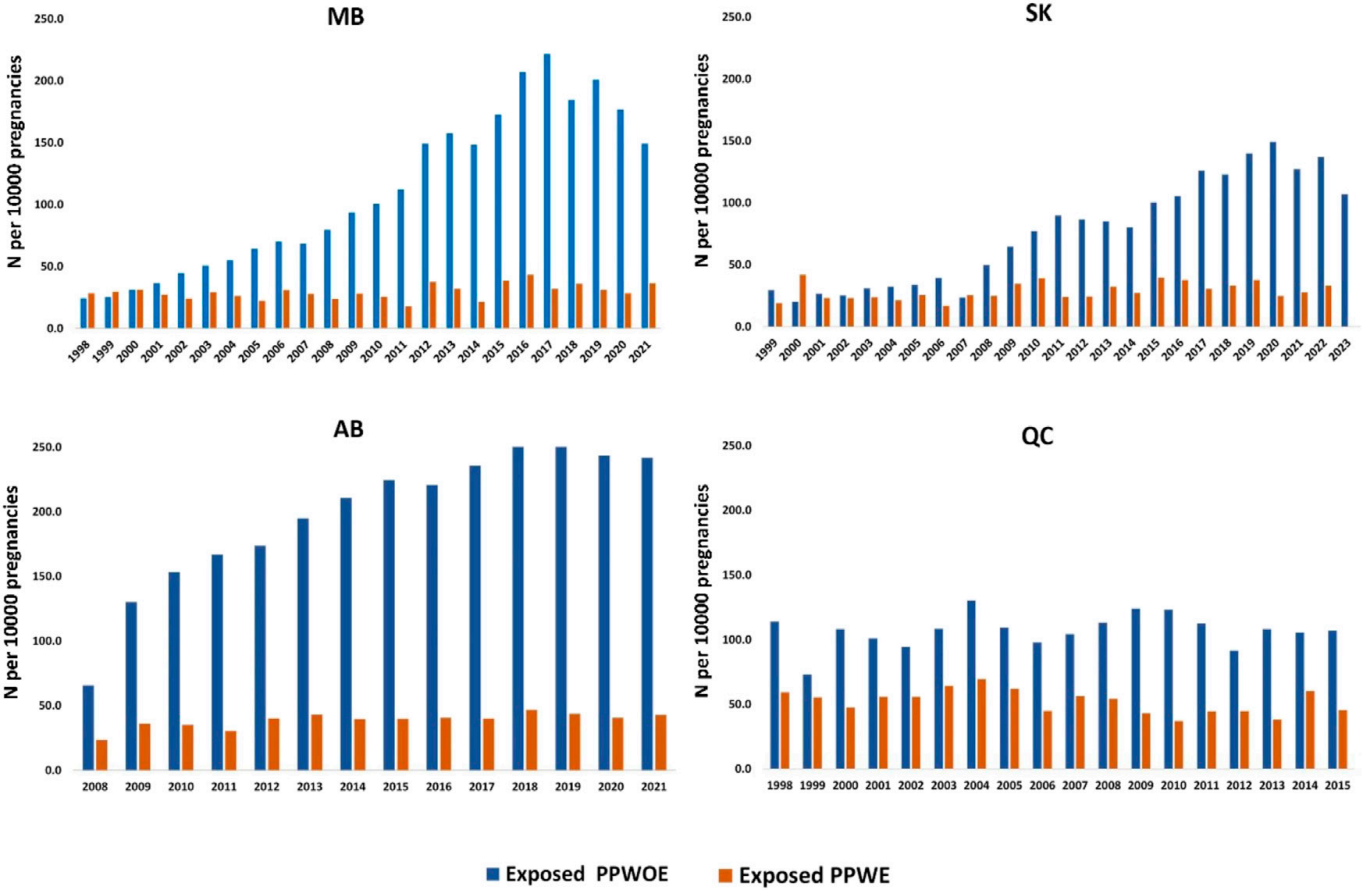
Annual utilization trend of ASMs among pregnant people with epilepsy (PPWE) and pregnant people without epilepsy (PPWOE). MB: Manitoba; SK: Saskatchewan; AB, Alberta; QC, Quebec. Numbers are reported per 10,000 pregnancies.

Figure 2 demonstrates the trends in ASM use in pregnant people with and without epilepsy. Linear regression analysis showed the number of PPWOE who used ASMs significantly increased in Manitoba (from 24.2 in 1998 to 149.1 per 10,000 pregnant people in 2021, *p* < 0.05), Saskatchewan (from 29.4 in 1999 to 107.0 per 10,000 in 2023, *p* < 0.05), Alberta (from 65.7 in 2008 to 241.7 per 10,000 in 2021, *p* < 0.05) and decrease in Quebec (from 113.9 in 1998 to 106.9 per 10,000 in 2015, *p* > 0.05. In Alberta, a significant rise in the number of PPWE exposed to ASMs was observed as well, starting from 23.6 in 2008 to 43.0 per 10,000 pregnant people in 2021 (*p* < 0.05). Conversely, the number of exposed PPWE in Quebec significantly decreased from 59.2 in 1998 to 45.5 per 10,000 pregnancies in 2015 (*p* < 0.05).

**FIGURE 3:**
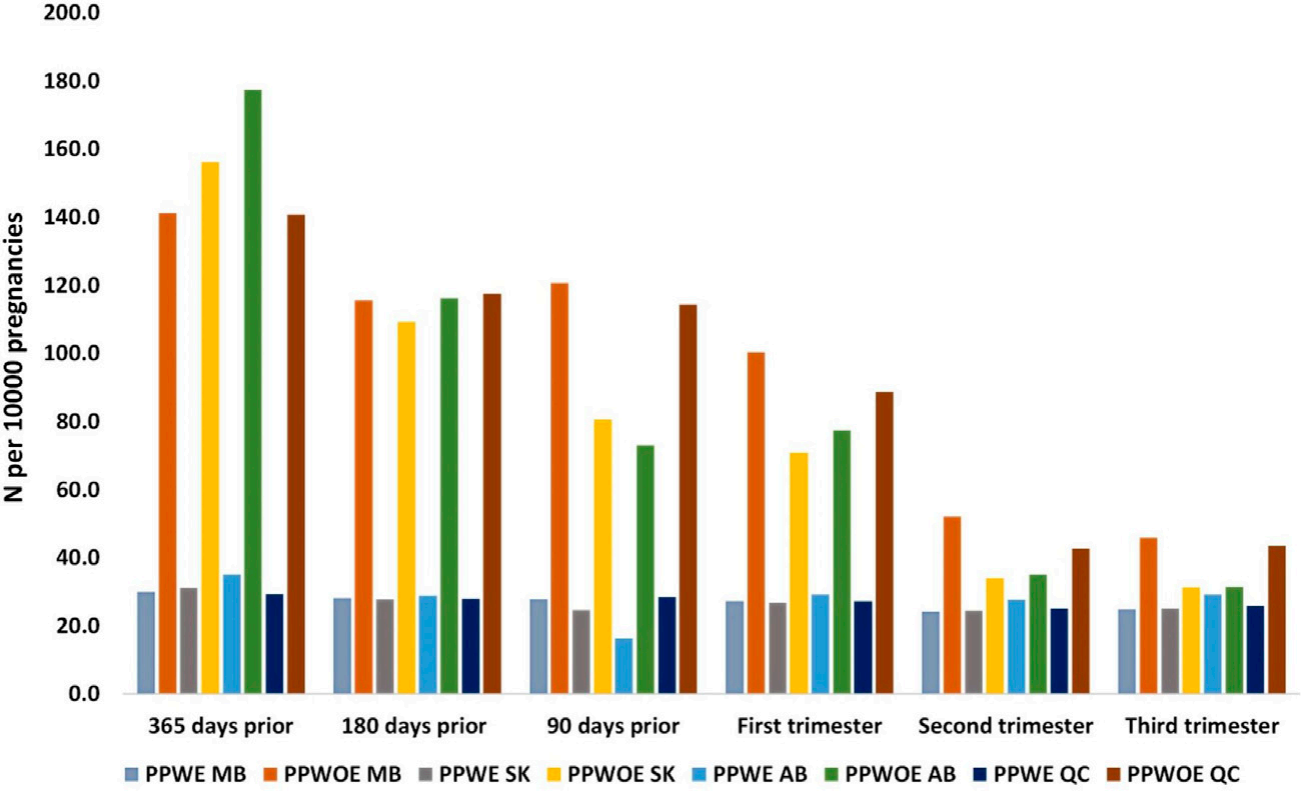
Number of pregnant people with (PPWE) and without epilepsy (PPWOE) who used ASMs in the pre-pregnancy period and each trimester per 10,000 pregnancies. MB, Manitoba; SK, Saskatchewan; AB, Alberta; QC, Quebec.

The number of pregnant people who used ASMs in the pre-pregnancy period and by trimester, in each province is shown in Figure 3. The use of ASMs in PPWE in four provinces did not change substantially during different periods. However, their use among PPWOE was highest 365 days prior to pregnancy and declined until the third trimester in all provinces.

**FIGURE 4:**
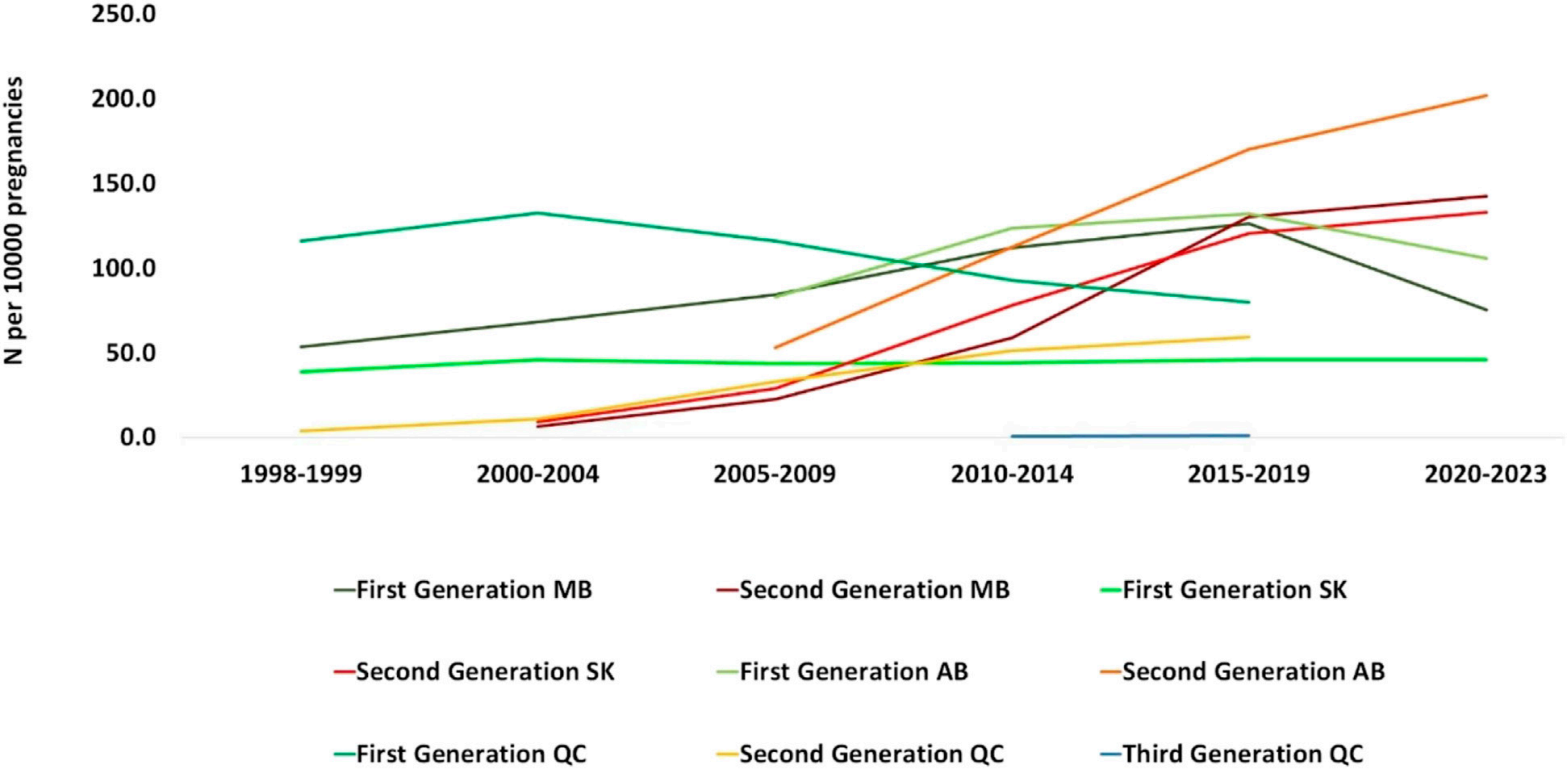
Number of pregnant people who utilized ASMs per 10,000 pregnancies by medication class (medications generation). MB, Manitoba; SK, Saskatchewan; AB, Alberta; QC, Quebec.

The utilization of ASMs by drug generation in periods between 1998 and 2023 is presented in Figure 4. In Manitoba, 53.2 people per 10,000 used first-generation ASMs during 1998–1999 and reached 68.1 during 2000–2004, while the number for second-generation ASM use was 6.1 per 10,000 pregnancies in 2000–2004. The numbers increased for both drug classes until they were used by the same number of people per 10,000 pregnancies in the 2015–2019 period. The utilization of second-generation ASMs kept rising in 2020–2021 and reached 142.2 people per 10,000 pregnancies while the use of first-generation ASMs decreased to 75.2. In Saskatchewan, the number of pregnant people using first-generation ASMs did not change over time. The utilization of second generation ASMs however, rose markedly from 8.9 person per 10,000 pregnancies in 2000–2004 to 133 in 2020–2023. For Alberta, data is available from 2008 to 2021. In 2008–2009, more pregnant people used first-generation ASMs compared to second-generation (82.8 vs. 52.7 per 10,000 pregnancies, respectively), whereas in 2020–2021, the number of pregnant people using second-generation ASMs was higher than those using first-generation ASMs (201.9 vs. 105.6 per 10,000 pregnancies, respectively). In Quebec, the majority of the population used first-generation in the 1998–1999 period and decreased over time until 2015 to 79.6 people per 10,000 pregnancies while second-generation drugs experienced an upward trend.

**FIGURE 5:**
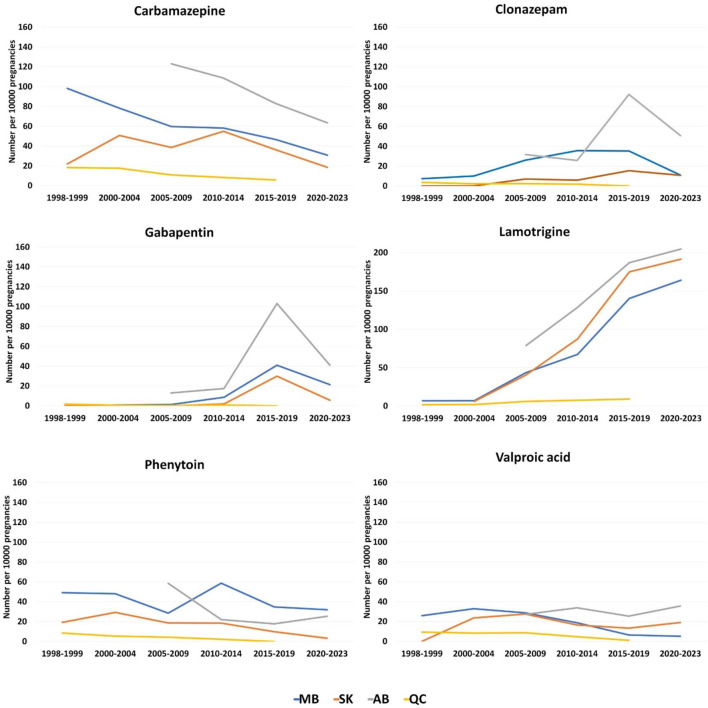
Total dispensations of top ASMs per 10000 pregnancies among pregnant people with epilepsy. **(A)** Carbamazepine; **(B)** Clonazepam; **(C)** Gabapentin; **(D)** Lamotrigine; **(E)** Phenytoin; **(F)** Valproic acid.

In the 1998–1999 period, carbamazepine was the most commonly dispensed ASM for PPWE which was 98.2, 21.9, and 18.3 per 10,000 pregnancies in Manitoba, Saskatchewan, and Quebec, respectively (Figure 5). It was also the most commonly dispensed ASM for PPWE at the beginning of the study in Alberta where the study started from 2008 (123.0 per 10,000 pregnancies during 2008–2009). However, the numbers dropped for carbamazepine until the 2015–2019 period, while lamotrigine had the highest number of prescriptions in this period (Manitoba: 140.3, Saskatchewan: 174.9, Alberta: 186.9, Quebec: 9.1). For Manitoba, Saskatchewan, and Alberta provinces that the data is also available for 2020–2023 period, the numbers of lamotrigine prescriptions continued to rise (164.0, 191.5 and 204.7 per 10,000 pregnancies, respectively). While levetiracetam was not among the most frequently prescribed ASMs in our dataset, we observed a significant rise in its utilization across all four provinces. In Manitoba, for instance, the use of levetiracetam increased from 0 in 1998–1999 to 120 per 10,000 pregnancies in 2021 among PPWE.

**FIGURE 6:**
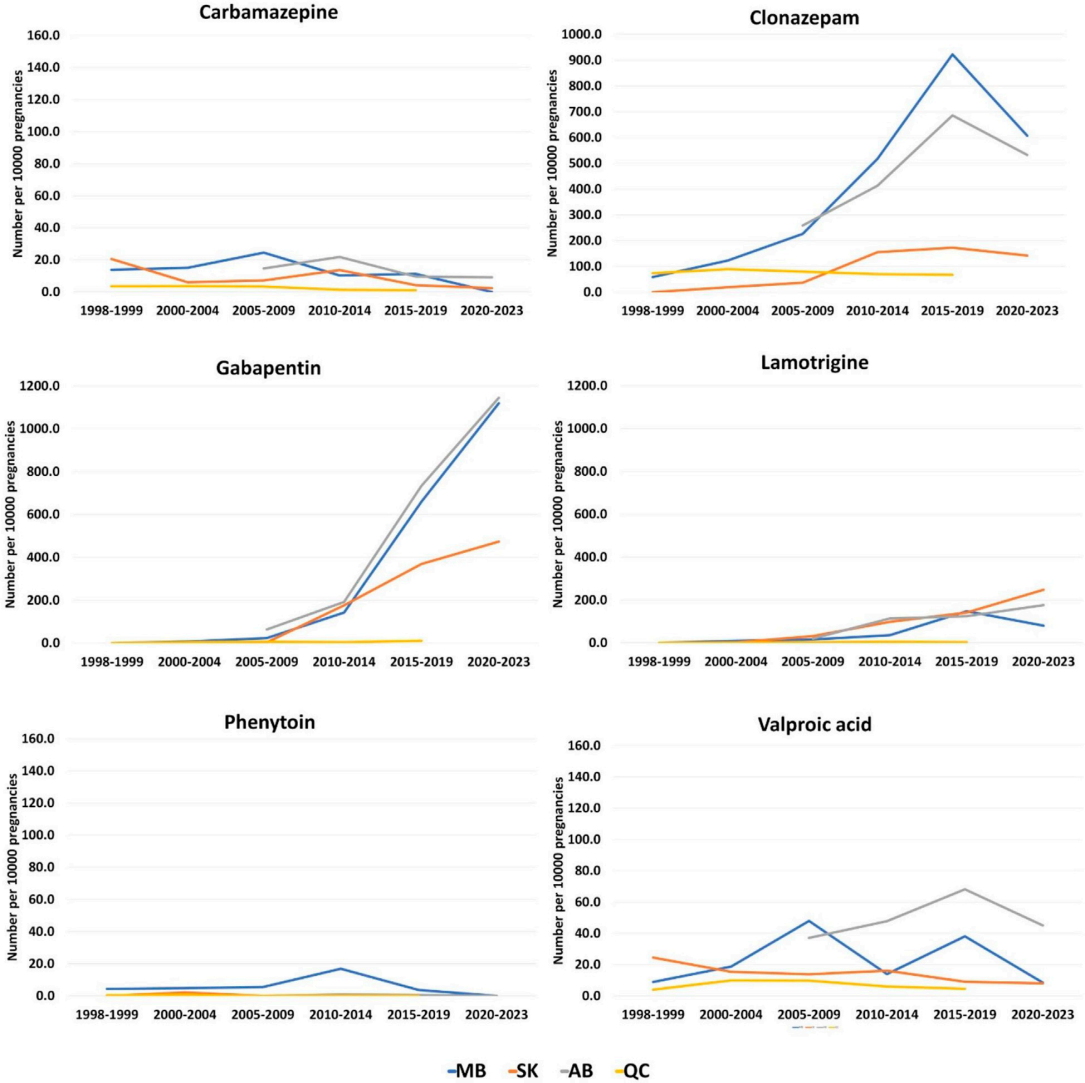
Total dispensations of top ASMs per 10000 pregnancies among pregnant people without epilepsy. **(A)** Carbamazepine; **(B)** Clonazepam; **(C)** Gabapentin; **(D)** Lamotrigine; **(E)** Phenytoin; **(F)** Valproic acid.

The prescription pattern for PPWOE was different. Clonazepam had the highest number of prescriptions during 1998–1999 in Manitoba (n = 58.5 per 10,000 pregnancies) and Quebec (n = 73.5 per 10,000 pregnancies) and with a sharp rise in the number of dispensations for Manitoba, it remained the most used ASM until 2019 in these two provinces (Figure 6). However, the pattern changed for Manitoba and gabapentin became the most prescribed ASM from 2020 to 2021 (n = 1,119.8 per 10,000 pregnancies). In Saskatchewan, valproic acid was the most prescribed ASM during 1998–1999 (n = 24.7 per 10,000 pregnancies). Whereas from the 2010–2014 period, the number of gabapentin prescriptions increased from 3.3 people per 10,000 pregnancies in the 2005–2009 period to 178.0 in the 2010–2014 period (Figure 6). The numbers kept rising for gabapentin until reaching 476.9 during the 2020–2023 period. In Alberta, clonazepam was the most prescribed ASM at the beginning of the study in the 2005–2009 period (n = 259 per 10,000 pregnancies). However, during 2015–2019 and 2020–2021, Gabapentin was the most prescribed drug in this province (734.2 and 1,145.1 per 10,000 pregnancies, respectively).

## 4 Discussion

In our study of over one million pregnancies with data ranging from 1998 to 2023 in four Canadian provinces, the utilization of ASMs during pregnancy varied between provinces and time periods.

Increasing trends were observed for the use of ASMs in Manitoba and Saskatchewan in PPWOE and among all pregnant people in Alberta, regardless of having been diagnosed with epilepsy.

In Manitoba, this rise was mostly due to the elevated prescription rates of clonazepam and gabapentin among PPWOE. In Saskatchewan, the rising trend in PPWOE was attributed to the increased dispensations of gabapentin, followed by lamotrigine and then clonazepam. In Alberta, the rise in ASM use among PPWOE was mainly due to the sharp elevation in the number of gabapentin dispensations, followed by clonazepam and lamotrigine, while the significant increase of ASM use in PPWE in this province was because of the elevated number of lamotrigine prescriptions. Alberta showed the highest increase in ASM use among both groups, while a decreased ASM use was observed in Quebec. This highlights the importance of considering provincial differences in prescribing patterns and utilization research.

The identified patterns are in line with the findings of recent similar studies. In a retrospective cohort study of 465,131 people of childbearing age exposed to ASMs in the United States from 2011 to 2017, the most significant linear increase in trends was observed with gabapentin (Lawal et al., 2023). In a multi-national study of five Nordic countries, the United States, and Australia from 2006 to 2016, the most commonly used ASM in pregnancy in the Nordic countries was lamotrigine and its use was increasing in all countries (Cohen et al., 2020). In the United States, however, clonazepam accounted for the most used ASM (Cohen et al., 2020). In another population-based study in the Netherlands from 1998 to 2019, lamotrigine use exhibited the highest increase of 24% among pregnant people (Houben et al., 2022).

In PPWOE, we observed a notable decline in the number of ASMs used from 365 days pre-pregnancy until the third trimester. Whereas in PPWE, the trends in ASM use did not change markedly. This could reflect the need for seizure control among PPWE and the fear of fetal harm among PPWOE, especially for medications like gabapentin and clonazepam (While clonazepam is a benzodiazepine with broader uses beyond epilepsy and gabapentin used for off-label indications such as neuropathic pain) (Lavu et al., 2023b; Madley-Dowd et al., 2024; Man et al., 2012). In a similar study, the total number of exposed pregnant people to ASMs in the pre-pregnancy periods and during pregnancy were compared and decreasing trends were witnessed in this population (Cohen et al., 2020).

Our results show that ASM utilization patterns among PPWE are changing towards ASMs with more favorable teratogenic profiles and avoiding drugs like valproic acid, which are consistently associated with congenital malformations (Tomson and Battino, 2012). We observed that in PPWE, lamotrigine use increased dramatically in all provinces. Among ASMs, lamotrigine is preferred as the first-line treatment option for people of childbearing age with epilepsy due to the least observed risk of congenital malformations and adverse birth outcomes (Veroniki et al., 2017). The significant rise in ASM use among PPWOE, particularly in Manitoba and Alberta, coupled with the shift from first-generation to second-generation ASMs, suggests a changing landscape in off-label ASM prescribing practices that warrants further investigation into the drivers of these trends and their potential implications for maternal and fetal health.

Declining use of valproic acid and phenytoin reflects increasing awareness of their teratogenic potential, changing clinical practice influenced by developing pharmacovigilance in pregnancy. This is in line with previously reported overall increasing trends in the utilization of the second-generation ASMs, especially lamotrigine and gabapentin. We also found a marked increase in the use of levetiracetam. This observation is concordant with reports of use among the general population. For instance, Liu et al. (2017), reported an increase in the use of levetiracetam in children and adolescents with epilepsy in (United States), while Fox et al. (2020), documented the same trend among elderly patients in (United States).

Striking differences in ASM use, particularly heavy clonazepam use among PPWOE, raise concerns about off-label prescribing and treatment of non-epileptic conditions in pregnancy. This suggests varying prescribing practices between specialties and highlights the need for improved education and guidelines. We did not have the capacity to analyze prescribers’ specialties in the current study. Further research is needed to identify predominant prescribers (general practitioners or specialists) to develop targeted interventions for optimizing prescribing practices, especially for managing anxiety and sleep disorders in pregnant patients without epilepsy.

Since ASMs introduction into the North America and Canadian market, the newer, safer ASMs have had a significant impact on prescribing patterns. Lamotrigine, for example, was first licensed in 1994 for use in epilepsy and much later, in 2003, for bipolar disorder, and was one of the first shifts to safer options. Currently, Levetiracetam approved in 2003 rapidly took the leading position because of an excellent safety profile. Pregabalin Approval for neuropathic pain in 2004 and generalized anxiety disorder in 2010 extended ASM use beyond epilepsy. These regulatory milestones are in concert with the secular trends observed in our study, demonstrating increased utilization of second-generation ASMs and their increased use in nonepileptic indications. Gabapentin utilization has been sharply increasing, primarily due to non-epilepsy indications, particularly for anxiety and pain. While the majority of research showed no major perinatal risks when it comes to its use during pregnancy, recent research has raised concerns regarding its association with major congenital malformations and neonatal intensive care unit admissions (Desrochers et al., 2024).

## 5 Strengths and limitations

This study leverages real-world data from medical records and prescription databases across four Canadian provinces over a 20-year period, providing a comprehensive and geographically diverse analysis of ASM utilization trends among pregnant individuals. The large sample size of over 1.3 million pregnancies enhances the statistical power and generalizability of our findings. Our longitudinal design allows for the observation of long-term trends, including the shift from older to newer generation ASMs, while distinguishing between use in pregnant people with and without epilepsy offers insights into off-label prescribing patterns.

Despite these strengths, some limitations should be acknowledged. First, the severity of epilepsy cases was not captured, medication exposure was based on dispensation records rather than confirmed consumption, and specific indications for ASM use in non-epilepsy conditions were not available. Additionally, the generalizability of findings for Quebec is limited due to data coverage of only 17% of pregnant people in the province. Nonetheless, the study provided valuable insights into ASM utilization trends during pregnancy in the province. (Bérard and Lacasse, 2009). Lastly, we did not report perinatal outcomes associated with ASM use, as this was out of the scope of the current study.

## 6 Conclusion

In conclusion, our comprehensive analysis of ASM utilization trends among pregnant individuals in four Canadian provinces over 2 decades reveals significant shifts in prescribing patterns. The study uncovered an escalation in ASM use among pregnant individuals in Manitoba, Saskatchewan, and Alberta, independent of an epilepsy diagnosis. The rise in prescriptions for newer generation ASMs, especially lamotrigine and levetiracetam, underscores a growing awareness of teratogenic risks and a shift towards safer treatment options. The increased use of newer ASMs, particularly among PPWOE, underlines the complex landscape of epilepsy and related disorders management during pregnancy. These findings highlight the need for continued pharmacovigilance, specialized care for pregnant individuals with epilepsy, and improved guidelines for managing comorbid conditions during pregnancy. The observed regional variations in prescribing practices further emphasize the importance of tailored and consistent healthcare approaches. Future studies should focus on the long-term outcomes associated with these changing prescription patterns and explore the factors driving off-label ASM use to further inform evidence-based practice in this vulnerable population.

## Data Availability

The data analyzed in this study is subject to the following licenses/restrictions: Due to privacy restrictions, the specific datasets analyzed for this article are not publicly accessible. The data originates from the Manitoba Population Research Data Repository, housed by the Manitoba Centre for Health Policy at the University of Manitoba. Requests to access these datasets should be directed to https://umanitoba.ca/manitoba-centre-for-health-policy/.
