# Supplementary material for: Trends of antiseizure medication utilization among pregnant people in four Canadian provinces from 1998 to 2023; a study from the Canadian mother-child cohort active surveillance initiative (CAMCCO)
Source: Front Pharmacol. 2024 Nov 12;15:1469552. doi: 10.3389/fphar.2024.1469552 (PMC11588457; doi:10.3389/fphar.2024.1469552)
Supplement: Supplementary file 1 [file Table1.DOCX]

**Supplementary Table 1: Appendix A: Table ATC codes for ASMs**

| No | Drug group | Basic code | Drug name |
| --- | --- | --- | --- |
| 1 | Barbiturates and derivatives: N03AA | N03AA01 | Methylphenobarbital, Mephobarbital |
|  |  | N03AA02 | Phenobarbital |
|  |  | N03AA03 | Primidone |
|  |  | N03AA04 | Barbexaclone |
|  |  | N03AA30 | Metharbital |
| 2 | Hydantoin derivatives: N03AB | N03AB01 | Ethotoin |
|  |  | N03AB02 | Phenytoin |
|  |  | N03AB03 | Amino(diphenylhydantoin) valeric acid |
|  |  | N03AB04 | Mephenytoin |
|  |  | N03AB05 | Fosphenytoin |
|  |  | N03AB54 | mephenytoin, combinations |
| 3 | Hydantoin derivatives:  N03AC | N03AC01 | Paramethadione |
|  |  | N03AC02 | Trimethadione |
|  |  | N03AC03 | Ethadione |
| 4 | Succinimide derivatives: N03AD | N03AD01 | Ethosuximide |
|  |  | N03AD02 | Phensuximide |
|  |  | N03AD03 | Mesuximide |
|  |  | N03AD51 | ethosuximide, combinations |
| 5 | Benzodiazepine derivatives: N03AE | N03AE01 | Clonazepam |
| 6 | Carboxamide derivatives: N03AF | N03AF01 | Carbamazepine |
|  |  | N03AF02 | Oxcarbazepine |
|  |  | N03AF03 | Rufinamide |
|  |  | N03AF04 | Eslicarbazepine |
| 7 | Fatty acid derivatives: N03AG | N03AG01 | Valproic acid |
|  |  | N03AG02 | Valpromide |
|  |  | N03AG03 | Aminobutyric acid |
|  |  | N03AG04 | Vigabatrin |
|  |  | N03AG05 | Progabide |
|  |  | N03AG06 | Tiagabine |
| 8 | Other epileptics: N03AX | N03AX03 | Sultiame, Sulthiame |
|  |  | N03AX07 | Phenacemide |
|  |  | N03AX09 | Lamotrigine |
|  |  | N03AX10 | Felbamate |
|  |  | N03AX11 | Topiramate |
|  |  | N03AX12 | Gabapentin |
|  |  | N03AX13 | Pheneturide |
|  |  | N03AX14 | Levetiracetam |
|  |  | N03AX15 | Zonisamide |
|  |  | N03AX16 | Pregabalin |
|  |  | N03AX17 | Stiripentol |
|  |  | N03AX18 | Lacosamide |
|  |  | N03AX19 | Carisbamate |
|  |  | N03AX21 | Retigabine |
|  |  | N03AX22 | Perampanel |
|  |  | N03AX23 | Brivaracetam |
|  |  | N03AX24 | Cannabidiol |
|  |  | N03AX25 | cenobamate |
|  |  | N03AX30 | Beclamide |

**Supplementary Table 2: Pregnancy Trimester Definition**

| Period of exposure | Definition |
| --- | --- |
| First trimester | 1st day of gestation to 14th week |
| Second trimester | > 14th week to < 25th week |
| Third trimester | ≥ 26th Week of pregnancy |
| Anytime during the pregnancy | 1st day of gestation – end of pregnancy |

**Supplementary Table 3: Comorbidities Definition**

| Diabetes | 3-years before conception, one or more hospitalizations with a diagnosis of diabetes (ICD-9-CM code 250 diabetes mellitus; ICD-10-CA codes E10-E14 diabetes mellitus), OR two or more physician histories with a diagnosis of diabetes (ICD) OR one or more prescriptions for antidiabetic medications (ATC code A10). |
| --- | --- |
| Mood and anxiety | At least one hospitalization with one year before conception,  -ICD-9-CM codes 296.2-296.8, 300, 309, 311; ICD-10-CA codes F31, F32, F33, F34.1, F38.0, F38.1, F40, F41, F42, F43.1, F43.2, F43.8, F44, F45.0, F45.1, F452, F48, F53.0, F68.0, F93.0, F99 OR One or more physician visits with a diagnosis for depressive disorder, affective psychoses, or adjustment reaction (ICD-9-CM codes 296, 309 or 311) OR Two or more physician visits with a diagnosis for anxiety disorders (ICD-9-CM code 300). |
| Personality disorders | one or more hospitalization with a diagnosis for personality disorders in one year before birth: ICD-9-CM code: 301; OR ICD-10-CA codes: F21, F34.0, F60, F61, F62, F68.1, F68.8 or F69; OR at least one physician visit with a diagnosis for personality disorders using ICD-9-CM code 301. |
| Schizophrenia | one or more hospitalization with a diagnosis for schizophrenia: ICD-9-CM code: 295 (schizophrenic disorders); OR ICD-10-CA codes: F20 (schizophrenia), F21 (schizotypal disorder), F23.2 (acute schizophrenia-like psychotic disorder), F25 (schizoaffective disorders); OR -one or more physician visits with a diagnosis for schizophrenia using ICD-9-CM code 295. |
| Hypertension | at least one hospital diagnosis for hypertension: (ICD-9-CM, 401: essential hypertension= 401-405 OR ICD-10-CA: I10-I14: essential (primary) hypertension); in the year before conception, OR -at least two ambulatory visit diagnoses: (ICD-9-CM codes: 401-405) in the year before conception (COHORTENTRY), OR - at least two dispensations for hypertension medication in the year before conception. |
| Pain | one year before conception: one or more hospitalization with a diagnosis of pain (ICD-9 code 338; ICD-10 R52 G89 G50) Neuralgia (ICD-9 729.2; ICD-10 M79.2) Migraine and Headaches (ICD-9 346 784; ICD-10 G43 R51) during the 1 year before conception, one or more codes in the physician data ICD-9 338,346,784. |
